# Maxillary Sinus Mucopyocele Mimicking a Radicular Cyst: A Case Report

**DOI:** 10.7759/cureus.110784

**Published:** 2026-06-13

**Authors:** Jinkimoni Singha, Sujata Byahatti, Praveena Tantradi, Renuka Ammanagi

**Affiliations:** 1 Oral Medicine and Radiology, Government Dental College Silchar, Silchar, IND; 2 Oral Medicine and Radiology, Maratha Mandal's Nathajirao G Halgekar Institute of Dental Sciences and Research Centre, Belgaum, IND

**Keywords:** dentistry related articles, maxillary sinus mucocele, oral histopathology, oral medicine and radiology, radicular cyst, swelling face

## Abstract

Maxillary sinus mucoceles are rare, benign, and expansile lesions that can lead to gradual enlargement and bony erosion. When a mucocele becomes infected, it is termed a mucopyocele or pyocele and can closely mimic odontogenic pathologies. We report the case of a 38-year-old male presenting with pain and swelling in the maxillary posterior region, mimicking a radicular cyst. Accurate diagnosis relies on a combination of symptoms, clinical evaluation, imaging, surgical exploration, and histological confirmation. This case highlights the clinical overlap between sinus mucopyoceles and odontogenic lesions, emphasizing the importance of a comprehensive diagnostic approach.

## Introduction

Paranasal sinus mucoceles represent benign, slow-growing lesions that develop within paranasal cavities. These lesions are characterized by accumulation of mucus secretions and are lined by epithelium. As the secretions accumulate, the resulting increase in internal pressure causes expansion, leading to thinning, erosion, and remodelling of the surrounding osseous walls [1]. The most common causes of paranasal sinus mucoceles are chronic infection, allergic rhinitis, allergic rhinosinusitis, previous surgical intervention, and maxillofacial trauma; however, the etiology may remain uncertain in some cases [2].

The frontal sinus is most commonly affected, followed by the ethmoid sinuses, whereas maxillary and sphenoid sinus involvement is less frequent [3]. When secondarily infected, a mucocele is termed a mucopyocele or pyocele.

Radicular cysts are inflammatory lesions of odontogenic origin, associated with non-vital teeth and may present as slow expansile growth. Radicular cysts associated with maxillary posterior teeth may occasionally be confused with a maxillary sinus mucocele due to their overlapping clinical features. Differentiating these conditions is important as their management strategies differ significantly.

We report a rare case of maxillary sinus mucopyocele that clinically mimicked a radicular cyst, highlighting the importance of comprehensive clinical, radiographic, and histopathological evaluation in distinguishing sinonasal lesions from odontogenic pathologies.

## Case presentation

A 38-year-old male reported to the Department of Oral Medicine and Radiology with a chief complaint of occasional pain in the upper left posterior teeth for the past year. The pain was intermittent in nature, moderate in intensity, and throbbing in type. The patient reported intermittent use of over-the-counter medications for symptomatic relief.

The patient reported the onset of swelling in the same region one month earlier, which gradually increased to its present size. There was no history of paresthesia, numbness, pus discharge, headache, or nasal obstruction. There was no relevant past medical history.

**Figure 1:**
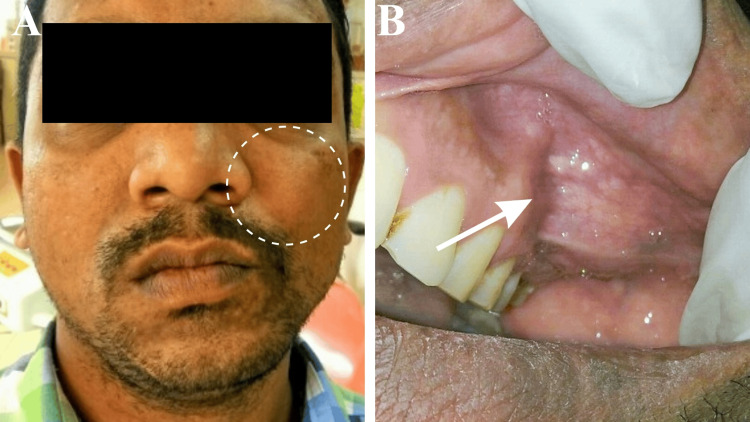
Clinical presentation of the lesion (A) Extraoral photograph showing diffuse swelling over the left middle third of the face; (B) Intraoral photograph showing swelling in the buccal vestibule

On extra-oral examination, a diffuse swelling was noted over the left middle one-third of the face (Figure 1). The overlying skin appeared normal. The swelling was non-tender and hard in consistency. Facial asymmetry was evident, with an oblique lip line and deviation of the chin toward the left side (Figure 1A).

On further inquiry, the patient reported a history of trauma to the left side of his face 12 years earlier, for which he didn't undergo any treatment. Following which, he developed facial asymmetry and occasional episodes of jaw locking that resolved spontaneously within a few seconds to minutes.

Intra-oral examination revealed a swelling in the buccal vestibule extending from the distal aspect of tooth 23 to the mesial aspect of tooth 28 in the upper posterior region (Figure 1B). The swelling was hard in consistency, non-tender, non-fluctuant with overlying mucosa that appeared normal. No palatal bulging was noted.

Hard tissue examination revealed root stumps associated with tooth 26. Other dental findings included decayed tooth 46, missing teeth 17, 16, 15, and root stumps with respect to tooth 14. Based on the clinical history and examination, a provisional diagnosis of radicular cyst in relation to tooth 26 was made. Antibioma was considered as a differential diagnosis.

**Figure 2:**
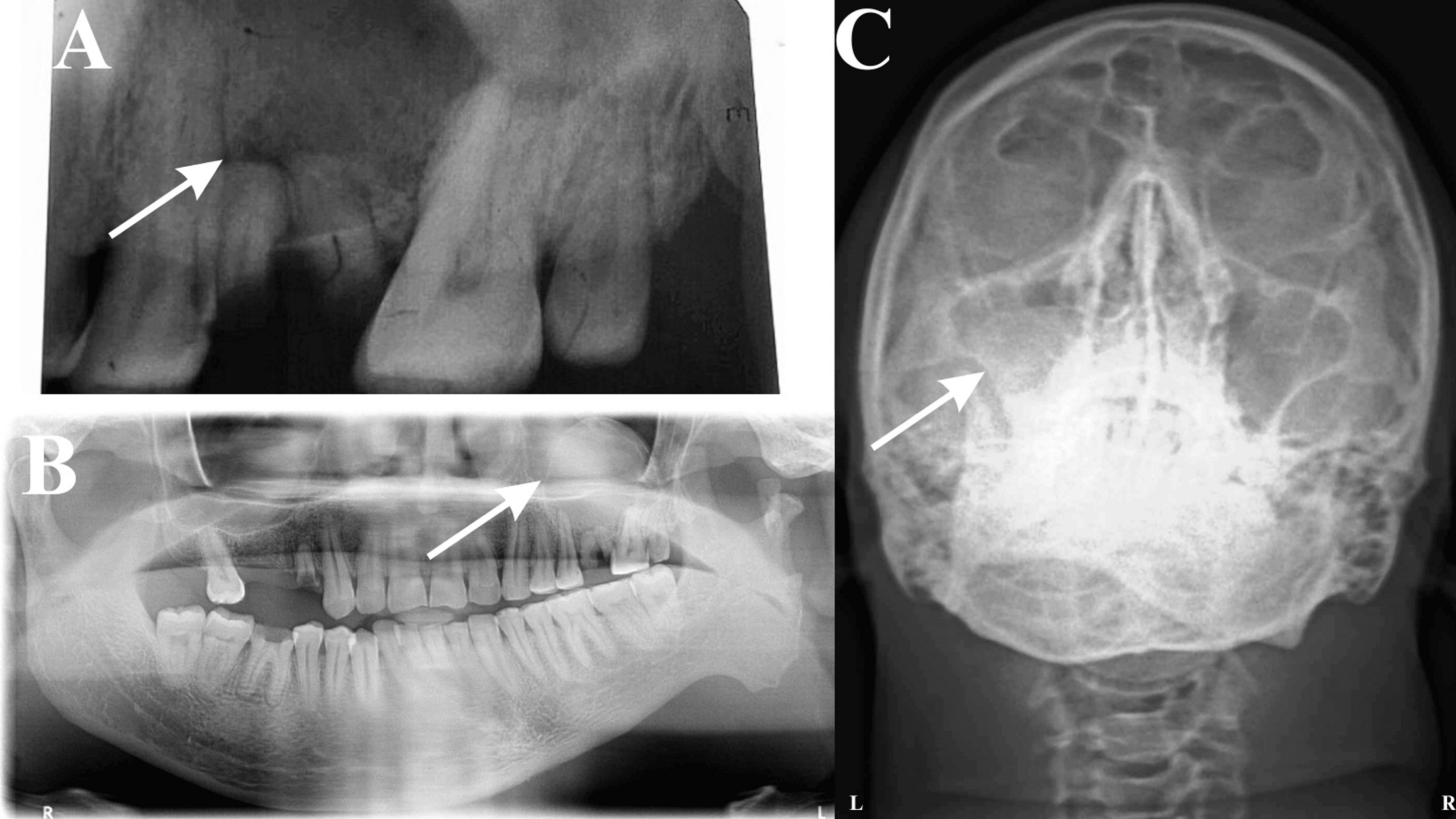
Various radiographic evaluations of the patient (A) Intraoral periapical radiograph showing an ill-defined radiolucency at the apex of tooth 26; (B) Panoramic radiograph demonstrating a hazy, radiopaque lesion occupying the left maxillary sinus; malunion of fractured condylar segments and elongated left coronoid process; (C) Waters' view showing diffuse opacification of the left maxillary sinus

The intraoral periapical radiograph in relation to tooth 26 revealed an ill-defined radiolucent lesion in the periapical area, suggestive of periapical abscess. A radiopaque line inferior to the lesion corresponded to the floor of the maxillary sinus (Figure 2A).

The panoramic radiograph and Waters' view revealed a diffuse, hazy radiopacity occupying a significant portion of the left maxillary sinus (Figures 2B, 2C). The borders were indistinct, and the lesion appeared to blend with the sinus cavity. These findings were suggestive of a maxillary sinus pathology. Additionally, the left condyle showed features suggestive of an old fracture with malunion of segments, along with elongation of the left coronoid process and a prominent left antegonial notch (Figure 2B).

**Figure 3:**
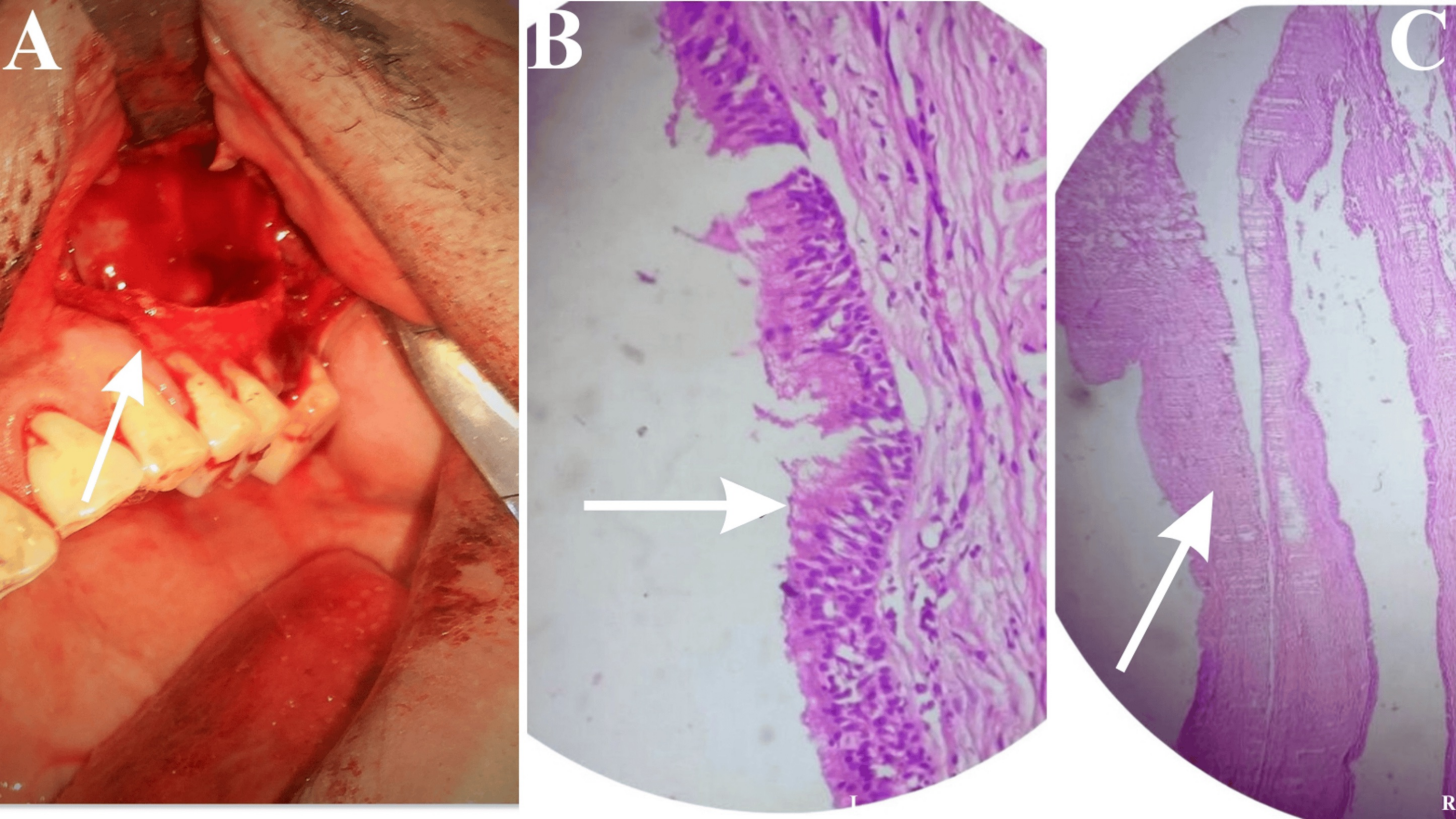
Surgical exposure and histological assessment (A) Surgical exposure and evacuation via the Caldwell-Luc approach; (B) Hematoxylin and Eosin (H&E) stained section showing pseudostratified ciliated columnar epithelium lining (40X); (C) H&E stained section showing pools of mucinous material within the lesion (10X)

Surgical exposure and evacuation via the Caldwell-Luc approach (Figure 3A) were performed, and abundant purulent mucus-like content was obtained from the cavity. The entire specimen was sent for histopathological evaluation.

Histopathological examination revealed pseudostratified ciliated columnar epithelium lining (Figure 3B), along with abundant mucin pools (Figure 3C) and underlying fibrous connective tissue with inflammatory infiltrate, which was suggestive of mucopyocele.

## Discussion

Paranasal sinus mucoceles were first described by Langenbeck in 1820, and he coined the term hydatids. In the year 1909, Rollet coined the name "mucocele" [4]. When a mucocele gets infected, it is known as mucopyocele or pyocele, and it is estimated to affect roughly 0.4% to 0.8% of the general population [5].

Paranasal sinus mucoceles are benign, grow slowly, and are characteristically lined by pseudostratified, ciliated columnar epithelium. They are produced by the accumulation of mucus secretions within the sinus cavity, secondary to obstruction of the sinus ostia. The obstruction could be due to chronic infection, allergic rhinitis, allergic rhinosinusitis, previous surgical intervention, maxillofacial trauma, and in some cases, the etiology may remain uncertain [2].

In the present case, a history of facial trauma 12 years prior was noted, which resulted in facial asymmetry and malunion of left condylar fracture segments. Although a direct causal relationship cannot be definitely established, prior trauma to his left side may have contributed to altered sinus drainage and ostial obstruction, thereby playing a role in the development of a mucocele.

The pathophysiological expansion of a mucocele is driven by continuous mucous retention, which increases internal pressure within the cavity and triggers an inflammatory cascade leading to release of cytokines, subsequently stimulating local fibroblasts to secrete prostaglandins and collagenase. These biochemical mediators stimulate osteoclastic activity and lead to bone resorption and expansion [6].

Mucoceles are predominantly diagnosed between the fourth and seventh decades of life with a minor male predilection [7]; they overwhelmingly favor the frontal sinus (60%-89%) and ethmoid sinus (8%-30%). Maxillary sinus involvement accounts for less than 5% and sphenoid sinus involvement is rarely seen [8].

Maxillary sinus mucoceles clinically manifest as painless swelling of the cheek or buccal cortical plate [7]. Clinical symptoms usually arise either due to progressive expansion or following secondary infection, resulting in a mucopyocele that manifests with pain [9]. In the present case, the patient reported a history of intermittent pain for one year, which may be due to the presence of a periapical lesion in relation to left maxillary tooth 26 also.

Depending on the direction of its expansion, a maxillary mucocele can cause diverse anatomical disruptions: medial expansion often displaces the inferior turbinate leading to nasal obstruction; superior expansion displaces the orbital content and induces visual disturbances; and downward expansion obliterates the buccal vestibule or even teeth mobility [7]. In the present case, obliteration of the buccal vestibule was noted.

Dental causes are unusual, but infection originating from a persistent carious deciduous maxillary molar tooth and ectopic third molar in the maxillary sinus have been reported to be precipitating factors [10,11]. The roots of the maxillary molars lie in close proximity to the floor of the maxillary sinus, facilitating the spread of odontogenic infection. In the present case, the patient had root stumps and associated periapical pathology in relation to the maxillary first molar, which may have contributed to the development of the lesion.

In the present case, a provisional diagnosis of a radicular cyst was made owing to the presence of root stumps in relation to the maxillary first molar 26 associated with a hard swelling in the buccal vestibule. Antibioma was considered in the differential diagnosis, given the patient’s history of intermittent use of over-the-counter medications for one year. However, radiographic evaluation revealed involvement of the maxillary sinus.

It was only through comprehensive panoramic and Waters' view radiography that a widespread hazy radiopacity of the left maxillary sinus was revealed. Radiographically, maxillary sinus mucoceles may present as homogeneous radiopaque lesions involving the maxillary sinus floor, or in advanced cases, the entire sinus cavity. An antral pseudocyst was also considered as a radiographic differential diagnosis, which presents as a dome-shaped radiopacity in the floor of the sinus; however, its non-expansile nature helped exclude it. Although computed tomography (CT) imaging was not performed in the present case, it is considered valuable in assessing the extent of the lesion, sinus wall integrity, and involvement of adjacent structures. 

Microscopically, mucoceles are lined by pseudostratified columnar ciliated epithelium with mucous cells. A definite diagnosis of a sinus mucopyocele cannot rely on clinical features alone. A correlation of patient symptoms, radiographic imaging, intraoperative findings during surgical exploration, and histopathological confirmation is necessary.

In this case, the Caldwell-Luc approach allowed for complete exposure, evacuation of mucoid material, and total removal of the lining.

Based on the review of the current literature, a limited number of cases have been reported where a maxillary sinus mucocele presented as an odontogenic lesion, thereby posing a significant diagnostic challenge [5,10,12]. Similar cases have described patients presenting with swelling in the maxillary posterior region along with obliteration of the buccal vestibule, findings that correlate with the clinical presentation observed in the present case [5,12].

## Conclusions

Maxillary sinus mucopyoceles are rare and may mimic odontogenic pathologies, such as radicular cysts, especially in the presence of a non-vital tooth. This case highlights the diagnostic challenge and emphasizes the importance of a multidisciplinary approach involving clinical, radiographic, surgical, and histopathological correlation for accurate diagnosis and effective management. A multidisciplinary approach is needed for the diagnosis and management of mucopyoceles.
